# Online mood monitoring in treatment-resistant depression: qualitative study of patients' perspectives in the NHS

**DOI:** 10.1192/bjb.2019.92

**Published:** 2020-04

**Authors:** Emma Incecik, Rachael W. Taylor, Beatrice Valentini, Stephani L. Hatch, John R. Geddes, Anthony J. Cleare, Lindsey Marwood

**Affiliations:** 1 Department of Psychological Medicine, Institute of Psychiatry, Psychology & Neuroscience, King's College London, UK; 2 National Institute for Health Research Biomedical Research Centre at South London & Maudsley NHS Foundation Trust and King's College London, UK; 3 Department of General Psychology, University of Padova, Italy; 4 Oxford Health NHS Foundation Trust, UK; 5 Department of Psychiatry, University of Oxford, UK; 6 South London and Maudsley NHS Foundation Trust, UK

**Keywords:** Treatment-resistant depression, mood monitoring, True Colours, qualitative research, major depression

## Abstract

**Aims and method:**

True Colours is an automated symptom monitoring programme used by National Health Service psychiatric services. This study explored whether patients with unipolar treatment-resistant depression (TRD) found this a useful addition to their treatment regimes. Semi-structured qualitative interviews were conducted with 21 patients with TRD, who had engaged in True Colours monitoring as part of the Lithium versus Quetiapine in Depression study. A thematic analysis was used to assess participant experiences of the system.

**Results:**

Six main themes emerged from the data, the most notable indicating that mood monitoring increased patients' insight into their disorder, but that subsequent behaviour change was absent.

**Clinical implications:**

Patients with TRD can benefit from mood monitoring via True Colours, making it a worthwhile addition to treatment. Further development of such systems and additional support may be required for patients with TRD to experience further benefits as reported by other patient groups.

Patients with treatment-resistant depression (TRD) can experience a more chronic and severe course of illness.^1^ These outcomes could be improved by online mood monitoring to facilitate treatment personalisation, and the evaluation of treatment efficacy.^2–4^ True Colours is an automated monitoring system developed by clinicians and currently used by psychiatric services in Oxford Health National Health Service (NHS) Foundation Trust, as well as by clinical trials across the country. Patients in Oxford Health are provided with access to True Colours by their clinical team at their or their clinicians request, whereas clinical trial teams can use the system to monitor participants and collect relevant outcome data. True Colours is also available via the Bipolar Disorder Research Network to patients with a diagnosis of bipolar disorder living in the UK.^5^ The system allows patients to self-monitor and self-manage symptoms^6^ by prompting them to complete personalised questionnaire assessments according to the time, frequency and mode of interaction selected. Patient responses are stored and displayed graphically through the interface, and are also made available to the patient's clinician or research team.

Adherence rates in patients with bipolar disorder are between 75 and 100%,^7,8^ and qualitative assessments indicated that patients with bipolar disorder find the system easy to use and a positive contributor to their clinical care and self-management.^9^ Comments on the flexibility of the system led to the development of a personalised questionnaire feature, which allows patients to add questions to their schedule.^10^ However, although the system is routinely used by patients with unipolar TRD, it is not clear whether they similarly benefit. Establishing the experience of this patient group is essential to maximising the clinical utility of this and other mood monitoring systems. This study aimed to qualitatively assess the perspectives of patients with unipolar TRD who used True Colours as part of the Lithium versus Quetiapine in Depression (LQD) study.^11^

## Method

Ethical approval was obtained from Cambridge South Research Ethics Committee (approval number 16/EE/0318). All participants provided written informed consent. This study has been carried out in accordance with the Declaration of Helsinki.

### Participants

LQD participants were invited to take part in this study between March 2017 and March 2018. This was a convenience sample, meaning that LQD participants attending an 8, 26 or 52 week follow-up assessment in that period were given the opportunity to participate in this additional study at the relevant follow-up assessment, conducted at a clinical research facility. Repeat interviews were not conducted. The full methodology of LQD has been reported.^11^ In brief, adult patients meeting the criteria for TRD were randomised to receive lithium or quetiapine augmentation and followed up over 12 months. As part of the study, participants were asked to complete two questionnaires weekly via True Colours to collect primary outcome data: the Quick Inventory of Depressive Symptomatology (QIDS-SR)^12^ and Work and Social Adjustment Scale (WSAS).^13^ Study-specific questions regarding medication were included to track dosage and adherence.

### Interview procedures

Participants took part in semi-structured interviews conducted by a researcher (full interview schedule is in the supplementary material, available at https://doi.org/10.1192/bjb.2019.92). Researchers were predominantly female and held a relevant undergraduate or master's degree. L.M. conducted training with all interviewers. Typically, the researchers had an existing relationship with the interviewees having conducted their prior LQD assessments. Interviews ceased once data saturation was reached, meaning no new emergent themes were being identified by the collection of additional data.^14^

### Data analysis

Interviews were audio-recorded, transcribed verbatim and checked by another researcher. All identifiable information was removed, and participants were assigned a code for reference.

A thematic analysis was conducted – a widely used method for identifying, analysing and reporting patterns within data – with steps recommended by Braun and Clarke.^15^ This included actively reading the entire data-set several times, systematically identifying and coding for key features, analysing codes and combining them to form broader themes. Themes were them reviewed and refined to ensure that they were appropriate in relation to the coded extracts, as well as the entire data-set. This led to the removal of some themes (e.g. owing to lack of supporting data), and the collapsing of others into one, with additional subthemes. Finally, themes were appropriately named, and evidence included for each in the present report. This approach is inductive (bottom-up), meaning the themes identified were data driven.^16^ Two researchers (E.I. and B.V.) conducted analyses independently, and discrepancies in the identified themes were discussed with L.M. and R.W.T. until resolved by consensus. The second rater (B.V.) did not conduct any qualitative interviews, minimising any potential bias.

## Results

A total of 26 individuals were invited to take part; 21 participated and five declined, primarily owing to fatigue (see Tables 1 and 2 for demographics and clinical characteristics). We generated 81 initial codes from the qualitative data and combined them to form six themes (see Table 3). Duration of interviews ranged from 3 to 17 min (mean 6.9 ± 3.3).

**Table 1:** Demographic characteristics of participants (*n* = 21)

Characteristic	
Age (years), mean (s.d.)	41.5 (15.2)
Gender, *n* (%)	
Female	8 (38)
Male	13 (62)
Employment status, *n* (%)	
Employed	10 (48)
Unemployed	7 (33)
Students	2 (10)
Retired	2 (10)
Education level, *n* (%)	
Primary education or less	1 (5)
Secondary education	3 (14)
College-level education or equivalent	5 (23)
Degree-level education/diploma	5 (23)
Postgraduate degree	7 (32)
NHS trust, *n* (%)	
South London & Maudsley NHS Foundation Trust	13 (62)
Northumberland, Tyne and Wear NHS Foundation Trust	3 (14)
Oxford Health NHS Foundation Trust	5 (24)

Percentages may not add up to 100 owing to rounding.

**Table 2:** Clinical characteristics of participants (*n* = 21)

Characteristic	
Total HRSD score,^a^ mean (s.d.)	22.3 (5.8)
Number of past episodes of depression, mean (s.d.)	4.1 (4.6)
Number of antidepressant treatment failures in current episode, *n* (%)	
2	13 (62)
3	3 (14)
4	4 (19)
5	1 (5)
Length of current episode in years, mean (s.d.)	8.4 (11.2)
Number of axis 1 comorbidities, *n* (%)	
0	4 (19)
1	5 (24)
>1	12 (57)
Overall mean % adherence to weekly True Colours data submission (s.d.)	72 (35)

HRSD, Hamilton Rating Scale for Depression. a. Hamilton M. A rating scale for depression. *J Neurol Neurosurg Psychiatry* 1960; **23**: 56–62. Percentages may not add up to 100 owing to rounding.

**Table 3:** Themes and subthemes

Theme	Subthemes
Insight	Anticipated insight (before using True Colours) Experienced insight Envisaged insight (gained through continued use of True Colours)
Absence of behaviour change	Not applicable
Improved clinical care	Using feedback during clinical appointments Valuing access to human support
Prioritisation	Organising time Motivation
Categorisation concerns	Discrepancy between experience and questionnaire response options Ambiguity and repetitiveness of questions
Interface features	Positive experiences Challenges Suggestions for improvement

### Insight

#### Anticipated insight (before using True Colours)

Participants frequently described how True Colours enabled greater understanding of illness. This outcome was anticipated by almost half of the participants upon hearing about the system.

‘I thought that, you know, it would give us something to go by, some kind of guideline, on, you know, where I am with my moods, or my behaviour and my sleeping and so forth’ (P6).

‘I had an expectation that it may be useful that you would have some form of er…information that you know, whether you're progressing or whether you're not in relation to your treatment, and in relation to how your illness is affecting you’ (P7).

#### Experienced insight

Regardless of whether it had been anticipated, 18 participants found that symptom monitoring enabled reflection. For some, viewing changes over time helped put their mood into perspective, particularly when feeling low. The process of completing questionnaire(s) appeared to play a key role in increasing awareness, and how mood linked to symptoms such as increased weight. Although many participants viewed this awareness positively, and one person reported a positive effect on their mood, a small number did not find it beneficial.

‘It's allowed me to monitor things ‘cause obviously certain weeks can be worse than others or better than others…so yeah it's been quite useful’ (P1)

‘Rather than thinking “I'm really depressed, I'm really depressed, I'm really depressed”, I'm able to kind of say “the score's the same” and actually, it's…your mood is the same…it's, you know, in your head that things are worse’ (P10).

‘You're looking at that [graphs] saying “Eee God I'm depressed” and it makes you feel even worse’ (P7).

#### Envisioned insight

This subtheme refers to insight that could be gained through continued use of True Colours. Six participants felt this could be possible, several of whom had not experienced significant mood fluctuations when using True Colours, but felt the graphs could help detect future changes. Others, who had not used the graphs discussed how they could offer future insight into mood patterns.

‘I would have thought that if I did use it [graphs], yes it would be useful…’'cause it would, you know, be able to tell me where the fluctuations are. With this said, I will make, um…endeavour to have a look in the next couple of weeks’ (P6).

‘I don't think it was originally that it would be particularly useful until I actually see how the changes are mapped on the graphs and stuff like that. So, the more interested I am, I think the more useful it'll be’ (P8).

‘I mean its early days but umm…but I think it [the graphs] will be useful in the future’ (P20).

### Absence of behaviour change

True Colours did not appear to affect the way in which most participants (*n* = 19) managed their depression. Because of the lack of changes reported, no subthemes were identified for this category. Participants recognised that the system could provide them with information, but did not see how this could translate to illness management.

‘Probably er…not err…not as much as manage it but be aware of it’ (P21).

‘No. Just…just it's informative. It doesn't change my lifestyle’ (P18).

### Improved clinical care

#### Using feedback during clinical appointments

Seven participants suggested the feedback provided via True Colours (i.e. graphs) could improve the efficiency of time spent with healthcare providers.

‘True Colours would be a way of keeping track of everything and also it would give me a visual representation to show medical professionals as opposed to just going well… I had a bad week 2 weeks ago. I can actually show them what happened as opposed to trying to remember it’ (P1).

‘I think for a professional that is dealing with your, or supporting you, I think that information I think may be useful’ (P7).

#### Valuing access to human support

For four participants, the awareness that they could be monitored by a professional who would understand their difficulties was a source of reassurance.

‘When I had a bad week then I emailed Tr…the…the link on True Colours…to say look, this is happening and…and it did help to know that somebody else understood what I was going through rather than me saying to somebody I'm feeling a bit…crap this week and they're just - oh…pooh poohing it really’ (P4).

‘Knowing that there is somebody out there that's monitoring me…which is nice’ (P3).

### Prioritisation

#### Organising time

Seven participants admitted that they struggled to schedule time for True Colours and often forgot to complete questionnaires.

‘There just isn't enough time in my day to do it’ (P5).

‘Sometimes I'll be working or something and I'll forget to do it’ (P1).

‘The only times I've kind of not done it is when I've kind of been really busy throughout the day’ (P10).

#### Motivation

Even setting aside the issue of time, over half of participants indicated that because of a lack of interest, not prioritising or viewing True Colours as useful and/or an inertia (related to their illness), it was difficult to engage consistently.

‘It depends where I am mentally on that particular day. Um…sometimes, do you know, I won't, I won't, won't be able to get out of bed to brush my teeth. And to be able, do you know, look onto your phone and fill out questionnaires, it's nigh on impossible’ (P6).

‘While I was sat there trying to psychoanalyse myself through True Colours I feel as though I could be doing something, achieving something that will possibly help me through this journey I am on’ (P5).

‘I think that you can spend too much time thinking about like erm being depressed, or the causes of depression, or how you're feeling, rather than living your life’ (P8).

### Categorisation concerns

#### Discrepancy between experience and questionnaire response options

Seven participants raised concerns regarding their ability to accurately summarise symptoms over the past week when there had been significant variability. They also felt there were not sufficient options to express experiences.

‘I couldn't categorise myself because my days are so…at the moment so mixed up’ (P5).

‘Umm…sometimes it's hard to put how you've been feeling or…kind of…getting an average…having a discrete box can be hard when you kind of want to do “well it's that point 5 or…”…ideally I'd say “it's just one between two points” rather than…a specific number’ (P15).

#### Ambiguity and repetitiveness of questions

Comments were also made regarding the wording of some questions, and how they were either difficult to understand, or similar to others. This left a small number of participants feeling unsure about how to respond.

‘There was one question on there which…um…seemed a bit…could be misconstrued’ (P2).

‘Yeah, yeah, like it's sometimes you feel it's getting rep…uh…repeating itself all the time’ (P3).

### Interface features

#### Positive experiences

Positive features relating to the interface were identified. Just over half of participants described the ease of logging on and completing questionnaire(s), and five participants commented on the usefulness of personalised weekly prompts.

‘Yeah…it's really easy…it's all… it's all laid out there for you so you just umm you know…tick whatever it is’ (P20).

‘I've had experienced nothing technical wise about it, nah, it's always been quite problem free’ (P7).

‘I think like it's good that there's a reminder…and that I could choose when it was. Umm…because it's like quite a convenient time for me just like in the evening to go on my phone, and it's quite quick to do the questionnaires…and like you can choose when that comes which I think's really good’ (P19).

#### Challenges

A variety of challenges relating to True Colours were similarly identified. Although participants were aware they could access response graphs, not all were using this feature. Four found the graphs difficult to interpret, and therefore not useful, and one commented on the lack of graphs for personalised questions. Further, 13 participants reported technical and interface issues, which affected their ability to complete questionnaire(s) and/or access feedback.

‘Like the symptom graph I don't quite get, and I don't quite understand how it works…erm…’cause I just see it as a load of blobs’ (P10).

‘I'd added like other questions just like for myself on there, but I wasn't able to see those on the graph, I could only see the study ones’ (P19).

‘It's not as good on the phone ‘cause you can't see it as properly as well as you can on the computer’ (P3).

‘I can't log in, I don't try anymore’ (P14).

Finally, two participants felt that human contact, rather than a technology-based approach, would more likely facilitate an open and honest sharing of information and aid recovery.

‘I think the only way you get to know things is by talking…I know that's not um…possible…but for people like me with my problem at my age we are not used to…um…baring our soul on a computer’ (P5).

‘I could fill a questionnaire and I could lie through my teeth, but I think you soon get caught out if you're sitting with a human being’ (P8).

#### Suggestions for improvement

Six participants volunteered information about ways in which the interface could be improved, including simplification of the questionnaire(s), and further personalisation options such as adding notes to questionnaire responses.

‘I still feel it can be simplified, to make it, make it a bit more user friendly. Realising that, you know, people using it may have various mental health issues, that might require, a bit more basis yes or no’ (P7).

‘Maybe if you could like… I don't know, like write notes at bottom or something, or like just for your own reference’ (P20).

‘Perhaps some way of changing the size of the text very easily would help, especially for people who aren't very computer literate’ (P15).

## Discussion

This study explored whether patients with unipolar TRD found True Colours, an online mood monitoring system used by NHS services, a useful addition to their treatment regimens. Our key findings were that mood monitoring enabled participants to feel that they had greater insight into their disorder, regardless of whether this was anticipated before use, but participants felt that their use of True Colours did not result in behaviour change. Many participants viewed their increased insight positively; but for some, spending time evaluating their symptoms was thought to contribute to a deterioration in mood. This aligns with the suggestion that the ability to identify and characterise one's mood state can predict positive affect, but a tendency to frequently scrutinise one's mood can predict negative affect and rumination.^17^

For most participants the perceived increase in insight was not associated with subsequent behaviour change. Neither completing the questionnaire(s) nor viewing the online graphs, which depicted their responses over time, led participants to make connections between patterns in their illness and their lifestyle choices. This is in contrast with research in patients with bipolar disorder, whereby monitoring via True Colours and other automated systems was associated with change in behaviour/improved self-management.^8,9^ This may be owing to the differing nature of TRD, which is not characterised by the same cyclic mood changes, and patients with unipolar TRD may require additional support for mood monitoring to inform behaviour change. It may be that patients with TRD who are undertaking therapies such as behavioural activation could benefit in this regard. The potentially unique needs of this patient group highlighted here would clearly benefit from further qualitative and quantitative research to fully understand how patients with TRD can benefit from this and other mood monitoring systems.

Although participants did not use True Colours for self-management, their responses suggested confidence that the system could improve clinical care by reducing reliance on their ability to accurately recall symptoms over time. The prospect of obtaining more contemporaneous data via this and other mood monitoring systems may improve our understanding of the course of major depressive disorder/TRD, and support the improvement of outcomes. Another key contributor to the enhanced clinical care theme was the belief that True Colours would give patients access to human support. This perception appeared to provide participants with a sense of support and reassurance, although this may have been inflated because of participants’ awareness that their adherence was monitored by the LQD study team. However, True Colours does facilitate real-time data sharing with clinicians in standard clinical practice, although this may not be the case with other mood monitoring systems, and whether or not data is monitored by a clinician should therefore be made clear to those who use any online mood monitoring platform.

Three key barriers to mood monitoring via True Colours were identified. First, participants indicated that it was difficult to find the time and/or motivation to engage consistently with the system. However, as discussed, LQD participants were required to complete two questionnaires each week as well as study-specific questions.^11^ In standard clinical practice, a manageable amount and frequency of use could be agreed between patient and clinician. Balancing the need to collect sufficient data while minimising the burden placed on patients is an important consideration and likely to be relevant to other online platforms.

Second, participants raised concerns about their ability to categorise experiences on standardised questionnaire(s). For some, there were clear discrepancies between what they had experienced and available response options. Others felt uncertain about question wording, reporting that they were difficult to understand or repetitive. Although these difficulties relate to the QIDS-SR and WSAS, and not the monitoring system itself, they indicate a need for the continued development of simplified self-report questionnaires to maximise the utility of online mood monitoring systems while maintaining the validity and reliability of assessments.

Finally, although participants had a positive experience with features of the interface (e.g. weekly prompts), a variety of issues were reported. Several participants chose not to view the graphs, and the majority of those who did had difficulty accessing or interpreting feedback. In addition, technical issues limited the ability of some to interact with the system. Although these graphical and technical concerns can be addressed, the finding that a small number of participants simply preferred human contact indicates that applications such as True Colours will not be acceptable to all, as is the case with any intervention.

There are limitations to this study. Convenience sampling was used, and although this is a widely used method of sampling in qualitative work, it may limit the transferability of the results to other settings, particularly as all participants were selected from a single clinical trial.^18^ Participants also varied in how long and to what extent they had used True Colours, making it difficult for some to comment on certain features (e.g. the graphs). The content and frequency of questionnaires was also protocolised according to the design of the wider clinical trial, although patients did have the flexibility to add additional questionnaires to their schedule. Therefore the experience of participants in the present study may therefore differ from those of patients using the system to support their usual care, and future investigation of online mood monitoring in a purely clinical setting would be of benefit. However, it is noted that the protocolised questionnaires included in this study are routinely used in clinical practice.

To our knowledge, this is the first study to explore the experiences of patients with TRD who use the True Colours mood monitoring system. Our findings are encouraging and suggest the system is reasonably well adhered to and provides an effective way of capturing outcomes. However further development is needed to improve the participant–system interface, and maximise the clinical utility of True Colours for this group. Another important step should be the examination of True Colours use in relation to treatment outcomes, to assess not only whether patients perceive the system to be beneficial, but also whether this translates to an improvement in empirical outcomes.

Although this qualitative study was conducted in a single sample taken from a clinical trial, the differences between the experiences of patients with unipolar TRD reported here and those of patients with bipolar disorder^9^ may have wider implications across settings and mood monitoring platforms. It is clear that online tools developed to support patient care are not ‘one size fits all’, and the experiences and preferences of individual patient groups must be accounted for during development if such tools are to provide the benefits intended.

## About the authors

**Emma Incecik** is an MSc student and Research Assistant at the Department of Psychological Medicine, Institute of Psychiatry, Psychology & Neuroscience, King's College London, UK. **Rachael W. Taylor** is a PhD student at the Department of Psychological Medicine, Institute of Psychiatry, Psychology & Neuroscience, King's College London; and National Institute for Health Research Biomedical Research Centre at South London & Maudsley NHS Foundation Trust and King's College London, UK. **Beatrice Valentini** is an MSc student at the Department of Psychological Medicine, Institute of Psychiatry, Psychology & Neuroscience, King's College London, UK; and Department of General Psychology, University of Padova, Italy. **Stephani L. Hatch** is a professor at the Department of Psychological Medicine, Institute of Psychiatry, Psychology & Neuroscience, King's College London, UK. **John R. Geddes** is a professor at Oxford Health NHS Foundation Trust; and the Department of Psychiatry, University of Oxford, UK. **Anthony J. Cleare** is a professor at the Department of Psychological Medicine, Institute of Psychiatry, Psychology & Neuroscience, King's College London; National Institute for Health Research Biomedical Research Centre at South London & Maudsley NHS Foundation Trust and King's College London; and South London and Maudsley NHS Foundation Trust, UK. **Lindsey Marwood** is Postdoctoral Clinical Trial Manager at the Department of Psychological Medicine, Institute of Psychiatry, Psychology & Neuroscience, King's College London; and South London and Maudsley NHS Foundation Trust, UK.

## Supplementary material

For supplementary material accompanying this paper visit https://doi.org/10.1192/bjb.2019.92. click here to view supplementary material
