# Supplementary material for: Online mood monitoring in treatment-resistant depression: qualitative study of patients' perspectives in the NHS
Source: BJPsych Bull. 2020 Apr;44(2):47–52. doi: 10.1192/bjb.2019.92 (PMC7283130; doi:10.1192/bjb.2019.92)
Supplement: Supplementary file 1 [file S2056469419000925sup001.docx]

**Supplementary Information for Online Mood Monitoring in Treatment Resistant Depression: A Qualitative Study of Patients’ Perspectives in the NHS**

**Qualitative Interview**

**Instructions to interviewer**: Please read the interview questions exactly as written and use the specific prompts listed under each question. General prompts can also be used as appropriate (e.g. what, why, how etc).

**Questions**:

**1. What were your expectations when you first heard about True Colours?**

*Prompt: Could you tell me a bit about that? (expectations)*
*Prompt: Useful tool or additional burden?*

**2. How have you found the process of completing the questionnaires on a weekly basis?**

**3. How has True Colours been useful for noticing changes in your mood?**

*Prompt: Can you tell me more about that? (experience of completing questionnaire and reflection - process of inputting)*

**4. True Colours allows you to track your mood over time on a line graph and a symptom graph. How useful have you found this feature?**

*Prompt: If helpful, how have they been helpful? If not helpful, can you think of any feedback that would be more helpful to you? (Feedback from the system)*
*Prompt: Useful, not useful? One more useful than the other?*

5. **How has True Colours influenced the way in which you manage your depression?**

*Prompt: Can you tell me more about this?*
*Prompt: The kind of things I am thinking of is lifestyle choices such as: exercise, eating patterns, working patterns, sleeping patterns. Also medication?*

6**. Can you describe any difficulties you have experienced in using True Colours?**
*Prompt: Any technical difficulties, motivation to respond to prompts (esp if feeling depressed), frequency of prompting*

**7. How would you feel about continuing to use True Colours once the study is over?**
*Prompt: Can you explain your reasons*
